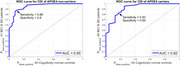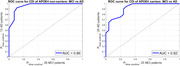# Physio‐markers based on predictive models of cerebral hemodynamics yield accurate diagnosis of cognitive impairment and differentiate MCI from AD

**DOI:** 10.1002/alz.091661

**Published:** 2025-01-09

**Authors:** Vasilis Marmarelis, Helena C Chui, Sandra A Billinger, Elizabeth B Joe, Dae Shin, Suhaib Hashem, Jasmin Rizko, Emily Hazen, Danilo Cardim, Rong Zhang

**Affiliations:** ^1^ Department of Biomedical Engineering, University of Southern California, Los Angeles, CA USA; ^2^ Department of Neurology, Keck School of Medicine, University of Southern California, Los Angeles, CA USA; ^3^ University of Kansas Alzheimer's Disease Research Center, Fairway, KS USA; ^4^ University of Southern California, Los Angeles, CA USA; ^5^ Kansas University Medical Center, Kansas City, KS USA; ^6^ UT Southwestern Medical Center, Dallas, TX USA; ^7^ UTSW Medical Center/IEEM, Dallas, TX USA

## Abstract

**Background:**

A multi‐center study in Los Angeles (USC), Kansas City (KUMC) and Dallas (UT‐SWMC) quantified via predictive modeling the dynamics of cerebral perfusion regulation (CO2 vasoreactivity and cerebral autoregulation) in MCI/AD patients and cognitively normal controls under resting conditions. The goal was to develop model‐based physio‐markers for accurate diagnosis of MCI and pre‐clinical AD, motivated by our previous findings of significant impairment of cerebral perfusion regulation in MCI and mild AD patients.

**Method:**

Continuous spontaneous changes in arterial blood pressure, end‐tidal CO2, cerebral blood flow velocity in middle cerebral arteries and cortical tissue oxygenation at lateral prefrontal cortex were recorded over two 6‐8 min sessions, separated by session of slow‐paced breathing (6 breaths/minute), in 53 MCI (28 APOE4 non‐carriers and 25 APOE4 carriers), 33 mild AD patients (13 APOE4 non‐carriers and 20 APOE4 carriers) and 74 age/sex‐matched cognitively normal controls (50 APOE4 non‐carriers and 24 APOE4 carriers). Using our novel modeling methodology, we obtained predictive dynamic models of cerebral perfusion regulation (dynamic CO2 vasoreactivity and cerebral autoregulation) that were used to compute a composite diagnostic index as physio‐marker of cerebral perfusion regulation dynamics under resting conditions for each participant. ROC curves were computed for differentiating MCI/AD patients from cognitively normal controls, and MCI from mild AD patients (separately for APOE4 carriers and non‐carriers).

**Result:**

The ROC curves for differentiating MCI/AD patients from controls are shown in Figure 1, for APOE4 carriers and non‐carriers separately, and had *Area Under the Curve (AUC) of 0.95 and 0.92*, respectively. The ROC curves for differentiating MCI from mild AD patients are shown in Figure 2, for APOE4 carriers and non‐carriers separately, and had *AUC of 0.96 and 0.92*, respectively. The p‐values of mean‐difference t‐tests for all four cases were *less than 10^‐8^* (highly significant).

**Conclusion:**

Quantitative analysis of cerebral perfusion regulation (dynamic CO2 vasoreactivity and cerebral autoregulation), using predictive dynamic modeling of cerebral hemodynamics under resting spontaneous conditions, yielded a Composite Diagnostic Index that differentiated MCI or AD patients from controls, and MCI from AD patients (p < 10^‐8^ for mean‐difference t‐test). The respective ROC curves had AUC in the range of 0.92‐0.96 (see Figures 1 & 2).